# The mental health impact of COVID-19 and lockdown-related stressors among adults in the UK

**DOI:** 10.1017/S0033291720005048

**Published:** 2020-12-07

**Authors:** Tarani Chandola, Meena Kumari, Cara L. Booker, Michaela Benzeval

**Affiliations:** 1 Department of Social Statistics and Manchester Institute for Collaborative Research on Ageing (MICRA), University of Manchester, Manchester M13 9PL, UK; 2 Institute for Social and Economic Research, University of Essex, Colchester CO4 3SQ, UK

**Keywords:** Mental health, Covid-19, stressors, unemployment, depression, UKHLS

## Abstract

**Background:**

The COVID-19 pandemic in the UK and subsequent lockdown may have affected the mental health of the population. This study examines whether there was an increase in the prevalence and incidence of common mental disorders (CMD) in the UK adult population during the first months of lockdown and whether changes in CMD were associated with stressors related to the pandemic and lockdown.

**Methods:**

Longitudinal data from the UK Household Longitudinal Study waves 10–11: 2019–2020 and waves 1–4 of the COVID-19 monthly surveys in April (*n* = 17 761) to July 2020 (*n* = 13 754), a representative sample of UK adult population, were analysed. CMD was measured using the 12-item General Health Questionnaire (GHQ-12) (cut-off >2). Changes in CMD were analysed in relation to COVID-19 and social stressors.

**Results:**

Around 29% of adults without CMD less than a year earlier had a CMD in April 2020. However, by July 2020, monthly incidence of CMD had reduced to 9%. Most employment, financial and psychological ‘shocks’ were at their highest levels in April and reduced steadily in later months. Despite the lifting of some lockdown conditions by July, stressors related to loneliness, unemployment, financial problems and domestic work continued to influence CMD.

**Conclusion:**

Some COVID-19 policy responses such as furloughing may have been effective in mitigating the increase in CMD for some groups of employees. Despite some reduction in levels of pandemic and lockdown-related stressors by the middle of 2020, loneliness and financial stressors remained key determinants of incidence in CMD among the UK adult population.

## Introduction

There have been large changes to social life in the UK during the COVID-19 pandemic. On 23 March 2020, a UK-wide lockdown was implemented (Dunn, Allen, Cameron, & Alderwick, 2020). People were not allowed to leave their home without a reasonable excuse. The strict lockdown conditions were relaxed in subsequent months with some regional variations. By 13 May, people could leave their homes for a limited set of activities and exercise more than once a day. By mid-June, most non-essential shops were allowed to reopen in England and small outdoor gatherings were allowed. Two different households could meet up either outside or indoors. From 4 July, restaurants, pubs and hairdressers in England were allowed to reopen. Towards the end of July, more social gatherings both indoors and outdoors were also allowed.

These severe and intense social restrictions, combined with the new disease, have resulted in an increase in potential stressors that could affect the mental health of the UK adult population. These stressors include those related to the disease itself, such as fear of catching the disease, or more indirect stressors due to changes in social life arising from disruptions to planned healthcare treatments because of the pandemic; the shutdown in the economy and the resulting increase in unemployment and financial stressors, new working patterns; additional home roles such as child care or home schooling; and feelings of loneliness due to lockdown conditions.

There is strong evidence that mental health and wellbeing in the UK worsened during the COVID-19 pandemic with the largest decline occurring in April (Public Health England, 2020). Data from the UK Household Longitudinal Survey (UKHLS) suggest that, among adults, mental distress [measured using the 12-item General Health Questionnaire (GHQ-12)] was 8.1% higher in April 2020 than it was between 2017 and 2019 (Xu & Banks, 2020). Mental distress (on the GHQ-12 scale) in April 2020 was 0.5 points higher than expected after taking into account trends in mental distress since 2013 (Pierce et al., 2020). In April 2020, over 30% of adults reported levels of mental distress indicative that treatment may be needed, compared to around 20% between 2017 and 2019 (Daly, Sutin, & Robinson, 2020). Evidence from other studies suggests that levels of anxiety, depression and stress were all higher than expected at the end of March and early April 2020 (Fancourt, Feifei, Wan Mak, & Steptoe, 2020a; Fancourt, Steptoe, & Bu, 2020b; Jia et al., 2020; Shevlin et al., 2020). They then show a moderate decrease in anxiety through April and May 2020, but not yet back to pre-pandemic levels.

There is also evidence that the COVID-19 pandemic has had a larger adverse impact on the mental health and wellbeing of some groups than others. Young adults and women have been more likely to report worse mental health and wellbeing during the pandemic than older adults and men (Xu & Banks, 2020). Women reported a larger increase in loneliness during the pandemic, as well as a greater degree of family and caring responsibilities, which could partially account for their higher levels of poor mental health compared to men (Etheridge & Spantig, 2020). Two studies found that adults living with children were more likely to report worse mental health than adults living without children (Kwong et al., 2020; Xue & McMunn, 2020). Adults with pre-existing mental health conditions reported higher levels of anxiety, depression and loneliness than adults without pre-existing mental health conditions, but there is no evidence to suggest that this gap has changed since the start of lockdown (Fancourt et al., 2020a, 2020b). One study found that adults who have had COVID-19-related symptoms were more likely to report high levels of mental distress and loneliness than adults who did not have such symptoms (Li & Wang, 2020).

Similar to pre-pandemic trends, adults with low household income or socioeconomic position reported more anxiety and depression than adults with higher household income or socioeconomic position (Bu, Steptoe, & Fancourt, 2020a, 2020b; Iob, Frank, Steptoe, & Fancourt, 2020; Wright, Steptoe, & Fancourt, 2020). Adults who were not in employment were more likely to report increasing levels of loneliness. Adults who experienced loss of income early in the lockdown reported higher levels of anxiety and mental distress (Bu et al., 2020a, 2020b; Wright et al., 2020). On the other hand, there is also evidence of higher mental distress among employed adults, as well as among adults with higher levels of education (Niedzwiedz et al., 2020; Pierce et al., 2020). The relationship between mental health, wellbeing and ethnicity is unclear with some studies reporting no significant association (Iob et al., 2020; Xu & Banks, 2020), while others suggest higher levels of mental distress among Asian than White British adults (Niedzwiedz et al., 2020; Pierce et al., 2020).

Most of these studies report on data from early stages of the pandemic and lockdown and have not examined how these COVID-19 and lockdown-related stressors changed as social restrictions were lifted. This is particularly important given the conflicting evidence around whether socioeconomically disadvantaged groups were at higher risk of poor mental health during the pandemic. Almost none of these studies examine whether changes in socioeconomic stressors correspond to changes in mental health. The effect of some stressors such as those related to unemployment and finances on mental health may have increased since the end of the first lockdown as businesses and employers struggled with the economic consequences of shutting the economy. On the other hand, some people may have become habituated to the lockdown conditions, have got used to the stressors of living with the pandemic, and may have recovered or become less vulnerable to developing a common mental disorder (CMD; Thompson & Spencer, 1966). Without longitudinal data that follow-up people's mental health and related stressors before and during the pandemic, it is hard to know to what extent the pandemic and lockdown has resulted in a ‘deep and lasting scar on the mental health of millions in this country’ (Mind, 2020).

### Our study had two research questions

RQ1. Has there been an increase in the prevalence and incidence of CMD problems in the UK adult population during the first few months of lockdown during the COVID-19 pandemic?

RQ2. Are the prevalence and incidence of CMD associated with any changes in stressors related to lockdown and the pandemic? Is there a difference between the associations of stressors with CMD in April 2020 compared to later months in 2020?

## Methods

### Data

This study uses longitudinal data from waves 10 and 11 (interim data) of the *Understanding Society*, the UK Household Longitudinal Study (UKHLS) and the April (*n* = 17 761), May (*n* = 14 811), June (*n* = 14 123) and July (*n* = 13 754) waves of the UKHLS COVID-19 2020 web survey. UKHLS is a nationally representative household panel study, which began in 2009 recruiting over 60 000 adults in 40 000 households (University of Essex, Institute for Social and Economic Research, NatCen Social Research, Kantar Public, 2019). It is a stratified clustered sample. Further details of the study design are available elsewhere (Buck, 2008). Interim data from waves 10 and 11 of UKHLS (with interviews in 2019 and 2020) have been released with the COVID-19 surveys to enable comparisons with more recently collected data compared to data collected in the previously available wave 9 (2017–2019). From April 2020, participants have been asked to complete a short web survey. This survey covers the changing impact of the pandemic on the welfare of UK individuals, families and wider communities. The response rate for the April 2020 COVID-19 web survey was just over 49% (Institute for Social and Economic Research, 2020). The response rate dropped 42% in the May survey and reduced to 39.2% by the July web survey. The web surveys were conducted in the last week of each month.

CMD was measured using the GHQ-12 designed to capture depressive and anxiety symptoms. The GHQ-12 is a widely used measure of non-psychotic psychological distress with excellent psychometric properties. The GHQ-12 has been validated against standardised clinical interviews and is considered as a unidimensional construct (Goldberg et al., 1997). Each item has four response categories on a Likert scale ranging from ‘not at all’ to ‘much more than usual’. For the analyses on incident CMD, we used the binary ‘GHQ-method’ of scoring (Goldberg & Williams, 1988) such that those responding to an item as ‘rather more’ or ‘much more’ than usual are scored as 1 and those responding as ‘not at all’ or ‘no more than usual’ are scored as 0. Scores are summed and range from 0 to 12. Respondents who score three or more on the GHQ-12 have probable CMD (Goldberg & Williams, 1988). We defined incident CMD as moving from a score of 2 or less in one wave to 3 or more in the next wave. ‘Recovery’ was defined as someone who had a CMD at the previous wave, but no longer had CMD at the current wave.

### Stressor variables

We conceptualised stressor variables in terms of social factors that are important for mental health that may have changed during the COVID-19 pandemic in the UK. Following the social determinants of mental health model (Allen, Balfour, Bell, & Marmot, 2014), these include COVID-19-specific stressors, and more indirect stressors arising from the UK lockdown conditions. COVID-19-specific stressors included reports of symptoms of COVID-19 (respondents were asked if they had ‘experienced symptoms that could be caused by COVID-19’) and reported testing for COVID-19 (no tests, tested negative/inconclusive/waiting for results and positive tests). Additional stressors included health treatment-related, family roles-related, economic, financial and psychological stressors.

Respondents were asked (every month) if their health treatments were cancelled or postponed, which, for those in urgent need, could be a source of stress.

Respondents were asked (every month) a series of questions on their current and previous employment status and working hours, and they were grouped into the following categories:

(a) The self-employed whose businesses were not affected by the pandemic (this was the reference category as the group that had the best working conditions);
(b) The self-employed whose businesses were directly affected by the pandemic in either April, May, June or July;
(c) Employees whose hours had not reduced in the past month(s);
(d) Employees who had been made unemployed or redundant or whose hours had reduced in the past month(s);
(e) Employees who were furloughed;
(f) Employees and the self-employed who were self-isolating or had care responsibilities;
(g) Those who were currently not in paid work.

Financial stressors included those who reported problems with paying their household bills in the April, May and June surveys. Respondents were also asked how they were managing financially and what their expectations were in a month's time.

Respondents were asked about a range of other potential stressors including working from home (every month), and hours spent on childcare and home schooling (in the April, May and June surveys). For the latter, on the basis of the clustering of responses, hours spent on childcare or home schooling in the last week were grouped into zero hours (if they had no children under the age of 18 or if they did not spend any time on these activities), 1–15 h a week and 16 or more hours a week.

Loneliness was measured (every month) by the question ‘In the last 4 weeks, how often did you feel lonely?’ at all the waves. Control variables for the regression models included age groups (in 5-year bands), sex, ethnicity, cohabitation with a partner, living with a child under the age of 5 years, educational qualifications, chronic or new health conditions and the time gap between the w10–11 survey and the April 2020 survey. The distributions of the control variables are shown in Appendix Table 1.

### Analysis plan

For RQ1, we calculated the prevalence, incidence and recovery rates from CMD for the April, May, June and July 2020 surveys. Incidence and recovery for the April survey were calculated from the w10–11 surveys. This was on average 9.7 months before the April survey and ranged from just under 19 months prior to just before the April survey. Thus, the incidence and recovery periods for the April survey cannot be compared with the later monthly surveys.

For RQ2, we analysed two types of regression models. The fixed-effects logistic regression models (fitted in STATA v14) examined how changes in the stressors affected changes in CMD. All time constant factors drop out of these models (such as age and ethnicity), thus eliminating time-invariant confounders of the association between stressors and CMD. However, these models cannot examine whether the associations of the stressors with CMD changed over the months. To analyse such time-varying associations, we used a random-effects (multilevel) logistic model clustering monthly observation periods (level 1) by participants (level 2) and the primary sampling unit (level 3). These multilevel models (fitted in MLwin v3.01) included the month of the survey as a set of dummy explanatory variables, in order to examine whether there were monthly differences in CMD. Interactions between month and all the potential stressor variables were analysed in order to examine whether the associations between stressors and CMD changed from month to month. All the multilevel models presented were ‘fully adjusted’ with all the potential stressor variables and control variables included simultaneously.

The multilevel models included inverse probability weights to take account of unequal selection probabilities into the study and differential non-response at each wave, including to the COIVD-19 monthly surveys. These weights ensure the results are reliable estimates and representative of the UK adult population living in private households using predictors that include basic demographics, household composition, economic variables and health variables, survey design variables and survey para data (Benzeval et al., 2020). The weights correct both for attrition from Understanding Society between wave 9 and relevant web survey wave, and non-response to that web survey (Institute for Social and Economic Research, 2020). Some of the stressor variables were only collected in specific months, resulting in two sets of analyses – one that included the April, May and June surveys (this included the domestic care and home schooling stressor variables) and the other that included the April, May and July surveys (this included the financial stressor variables).

## Results

**Fig. 1.**
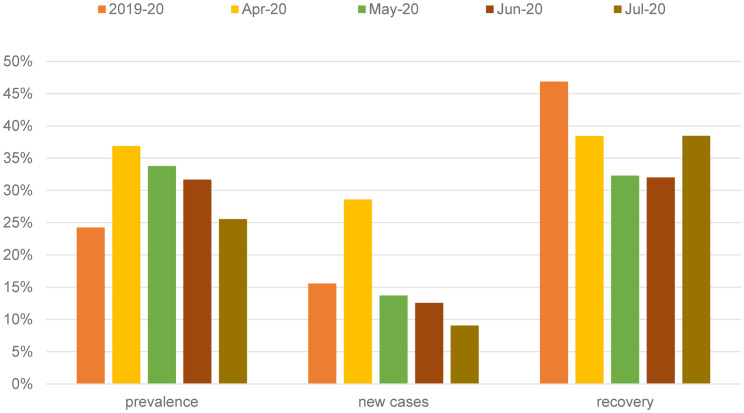
Prevalence, new cases and recovery from common mental disorder – UKHLS COVID-19 survey.

The trends in CMD (CMD prevalence, new cases and recovery) in the COVID-19 monthly surveys are shown in Fig. 1. The prevalence of CMD in the UK adult population was 37.2% in April 2020 (Table 1). This decreased steadily each month and by July the prevalence was 25.8%. New cases of CMD in April among participants who did not report any CMD in the previous wave (on average about 9.7 months before) was 28.6%. In contrast, the monthly incident rate of CMD was much lower in subsequent months, decreasing by more than a third of the April level by July. Recovery from CMD in April relative to the previous wave (just under 10 months before) was 38.4%. The monthly recovery rate decreased to 32 in May and June, but by July, the recovery rate was similar to the April levels.

**Table 1.** Distribution of key variables by survey month: UKHLS-COVID-19 monthly surveys (weighted estimates)

	April (%)	April (*n*)	May (%)	May (*n*)	June (%)	June (*n*)	July (%)	July (*n*)
CMD prevalence								
No CMD	62.8	7883	65.3	7574	67.9	7524	74.2	8072
Common mental disorder (CMD)	37.2	4677	34.7	4017	32.1	3550	25.8	2804
CMD new cases								
No CMD	71.4	6342	86.3	5647	87.4	5683	90.9	5914
CMD (new cases)	28.6	2540	13.7	897	12.6	816	9.1	590
CMD recovery								
Recovery (no more CMD)	38.4	1145	32.3	1250	32.0	1094	38.5	1148
CMD	61.6	1833	67.7	2623	68.0	2324	61.5	1835
COVID-19 symptoms								
At least one	11.8	1645	3.6	432	2.3	264	2.3	257
None	88.2	12 322	96.4	11 563	97.7	11 233	97.7	10 965
COVID-19 test								
No test	99.0	13 827	96.1	11 530	95.3	10 953	94.1	10 563
Negative or inconclusive test	0.9	126	3.7	447	4.6	529	5.7	637
Positive test	0.1	16	0.2	21	0.1	15	0.2	22
Health treatments								
No ongoing treatment	79.4	10 932	84.3	10 087	84.0	9611	84.6	9465
Treatments cancelled/postponed	12.9	1777	9.9	1189	8.5	970	6.7	744
I cancelled treatment	2.0	282	1.4	170	1.3	154	1.0	113
Treatments as scheduled	5.6	773	4.3	516	6.2	706	7.7	862
Employment status								
Slf-emplyd: no change in hours	2.6	337	6.0	707	6.1	693	6.0	669
Slf-emplyd: affected by COVID	3.6	481	1.0	119	0.5	61	0.6	64
Employee: no hours affected	33.4	4406	47.5	5637	49.6	5632	50.0	5584
Unempld/redund/reduce hrs	3.0	402	1.4	167	0.6	71	1.2	137
Furloughed	14.4	1894	4.2	501	2.3	266	1.4	161
In isolation or caring	4.2	560	1.1	129	0.9	99	0.7	81
Not in work	38.8	5115	38.9	4614	39.9	4530	40.0	4461
How often working from home								
Always	16.5	2302	17.8	2134	16.7	1905	14.9	1657
Often	3.2	442	3.9	465	4.0	455	4.5	500
Sometimes	3.5	491	4.5	541	4.4	503	5.1	569
Never	15.4	2150	17.7	2115	20.9	2391	24.2	2701
No paid work hours	61.4	8561	56.1	6703	54.1	6184	51.3	5723
How often you feel lonely								
Hardly ever	60.7	8207	60.0	7165	59.0	6748	61.0	6813
Some of the time	30.5	4125	31.3	3734	33.1	3792	32.3	3603
Often	8.8	1188	8.8	1048	7.9	898	6.7	753
Hrs/week childcare/home school								
No children under 18/0 h	80.8	11 171	79.4	9470	80.8	9190		
1–15 h/week	10.0	1383	11.9	1416	12.4	1409		
16 h or more/week	9.2	1272	8.7	1036	6.8	777		
Problems paying bills								
Up to date	93.5	12 109	92.7	10 858			93.2	10 257
Behind with some bills	6.0	781	6.9	814			6.3	697
Behind with all bills	0.5	65	0.4	47			0.4	48
Subjective financial situation								
Living comfortably	31.8	4136	30.7	3603			26.9	2960
Doing alright	43.1	5603	44.6	5245			47.1	5197
Just about getting by	18.4	2399	18.6	2185			19.4	2134
Finding it quite difficult	4.7	615	4.5	532			5.2	573
Finding it very difficult	1.9	252	1.6	192			1.4	158
Future expectation of finances								
Better off	7.7	998	8.3	977			9.6	1064
Worse off than now	16.4	2133	11.5	1345			10.0	1101
About the same	75.9	9862	80.2	9423			80.4	8866

The decrease in the prevalence of CMD from April to July was mirrored by a decrease in the levels of stressors over the same period (Table 1). Reports of having COVID-19-related symptoms were 11.8% in April but incidence had declined to only 2.3% in July. Unsurprisingly, more people reported (ever) taking the COVID-19 test in July compared to April but rates of positive tests for the virus were very low throughout the period. Nearly 15% of the adult population with limiting conditions reported either cancelling NHS-related treatments or having their treatments cancelled or postponed in April. By July, this figure had reduced to 7.7%. In terms of employment-related changes, there was a marked decrease in the proportion of the self-employed who reported their businesses had been negatively affected by COVID-related restrictions from April (3.6%) to July (0.6%). Over the same period, the proportion of employees who were unemployed or whose hours were reduced fell from 3% to 1.2%. Rates of those in furlough or those in isolation due to sickness or caring responsibilities also fell considerably compared to April levels. There was a small increase in the proportion of economically inactive people from April to July. There were relatively more people working from home in April compared to July. Rates of ‘often feeling lonely’ fell from 8.8% in April to 6.7% in July. The percentage of respondents who spent more than 16 h a week on childcare or home schooling reduced from April to June, although there was a small increase in the proportion who spent 1–15 h/week on those tasks over the same period. Problems with paying bills were relatively constant from April to July, but there was a small decrease in the proportion of adults who reported they were finding it very difficult in terms of their current finances (1.9–1.4%) and a decrease in the proportion whose future expectations of finances was worse off than their current situation (16.4–10%).

Table 2 displays the fixed-effects coefficients of CMD regressed on potential stressors that changed over two periods – in the April, May and June surveys (without the financial variables as these questions were not asked in the June survey), and in the April, May and July surveys (without the childcare/home schooling hours variables which were not asked in July). Changes in reports of loneliness were the biggest predictor of an increase in CMD – respondents who reported often feeling lonely were 11 times (95% CI 8.5–14.3) more likely to have CMD in the April to June surveys, and 16 times (95% CI 12.1–21.0) more likely to have a CMD in the April to July surveys. Other stressors that were associated with developing a CMD in both survey periods were reporting COVID-19 symptoms (OR ranging from 1.6 to 2.0) and always working from home (those who never worked from home were 0.5–0.7 times less likely to develop a CMD compared to those who always worked from home). People who had no planned healthcare treatments were less likely to develop a CMD in both periods. The self-employed whose businesses were negatively impacted by COVID-19 were more likely to develop a CMD compared to their peers whose businesses were not affected by COVID-19. Furthermore by July, employees who became unemployed, or were made redundant or whose work hours were reduced were over two times as likely to develop a CMD compared to the self-employed whose businesses were not affected by COVID-19. Adults who were spending 16 h or more a week on childcare on home schooling were about 1.4 times (95% CI 1.0–1.9) more likely to develop a CMD compared to those who had no children or did not spend any time on childcare. Adults who were finding it quite or very difficult financially were 2.4 times (95% CI 1.7–3.3) more likely to develop a CMD compared to those who were living comfortably. Similarly, adults who expected their future finances to be worse off than now were 1.6 times (95% CI 1.3–1.9) more likely to develop a CMD compared to those who expected to be better off. Having a COVID-19 test (but not a positive test result) was associated with lower odds of developing a CMD in the April–July surveys compared to adults who did not have a COVID-19 test (Table 2).

**Table 2.** Fixed-effects odds ratios (95% CI) of common mental disorder regressed on potential stressors: UKHLS-COVID-19 monthly surveys

	April-May-June	April-May-July
*n* (observations)	12 166	12 765
*n* (individuals)	4264	4477
Reported COVID-19 symptoms (ref: none)		
At least one	**1.59 (1.33–1.89)**	**1.97 (1.64–2.36)**
Reported COVID-19 test (ref: no test)		
Tested for COVID-19	0.93 (0.74–1.17)	**0.74 (0.60–0.91)**
Tested positive	1.60 (0.64–4.02)	1.05 (0.37–2.95)
How often working from home (ref: always)		
Often	0.85 (0.65–1.11)	**0.76 (0.60–0.97)**
Sometimes	0.83 (0.62–1.12)	**0.54 (0.41–0.71)**
Never	**0.69 (0.51–0.94)**	**0.46 (0.35–0.60)**
No paid work hours	1.12 (0.85–1.46)	**0.79 (0.62–1.00)**
Health treatments (ref: no treatments planned)		
Treatments cancelled/postponed	1.17 (0.96–1.41)	**1.24 (1.03–1.49)**
I cancelled treatments	1.36 (0.95–1.94)	**1.50 (1.04–2.16)**
Alternative treatment/scheduled	**1.32 (1.08–1.61)**	**1.28 (1.06–1.55)**
Employment status (ref: self-employed not affected)		
Self-employed: -vely impacted by COVID	**1.64 (1.20–2.25)**	**1.43 (1.03–1.99)**
Employee: hrs not affected	0.99 (0.65–1.50)	1.17 (0.77–1.80)
Employee: redundant/unemp/reduced hrs	1.23 (0.75–2.04)	**2.08 (1.25–3.48)**
Employee: furloughed	1.12 (0.72–1.75)	1.50 (0.95–2.38)
Self-isolating/caring	1.27 (0.85–1.91)	**1.67 (1.09–2.54)**
Not in work Jan/Feb	1.19 (0.74–1.90)	**1.75 (1.08–2.86)**
How often feel lonely (ref: hardly/never)		
Some of the time	**3.13 (2.78–3.53)**	**3.10 (2.76–3.49)**
Often	**11.05 (8.51–14.34)**	**15.97 (12.1–21.01)**
Hrs/week on childcare/home school (ref: no child or <18/0 h)		
1–15 h/week	1.21 (0.93–1.58)	
16 h or more/week	**1.37 (1.02–1.86)**	
Problems paying bills (ref: no problems)		
Behind with some bills		1.35 (0.98–1.86)
Behind with all bills		2.25 (0.53–9.64)
Subjective financial situation (ref: living comfortably)		
Doing alright		0.98 (0.85–1.14)
Just about getting by		**1.42 (1.14–1.77)**
Finding it quite/very difficult		**2.37 (1.68–3.34)**
Future expectation finances (ref: better off)		
Worse off than now		**1.55 (1.26–1.90)**
Or about the same?		0.83 (0.7–0.98)

Bold figures denote *p* < 0.05.

**Fig. 2.**
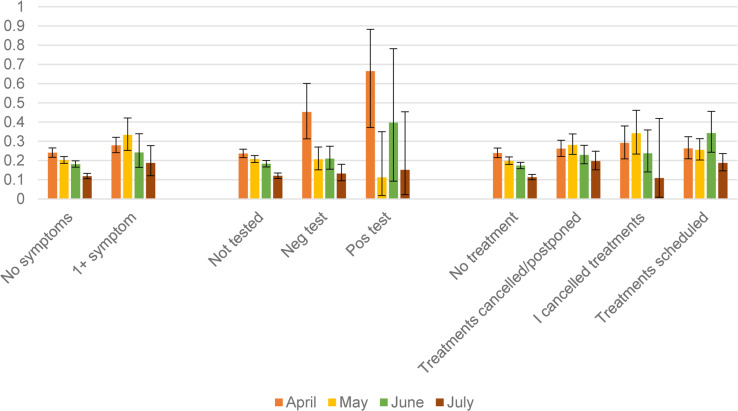
Predicted probabilities (and 95% CI) of common mental disorder: estimates taken from April–July 1 (Appendix Table 2).

**Fig. 3.**
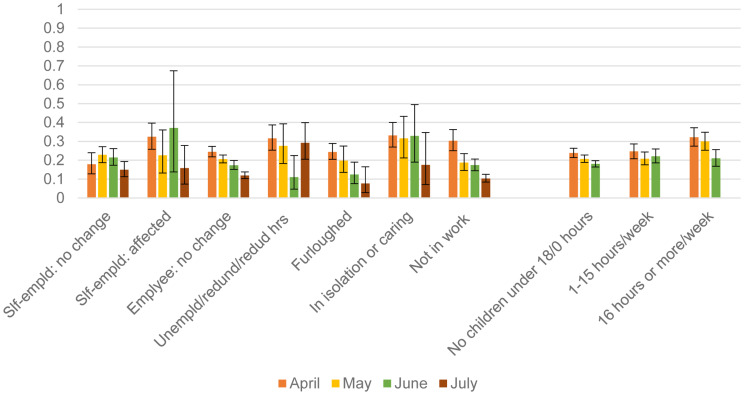
Predicted probabilities (and 95% CI) of common mental disorder: estimates taken from April–July and April–June models (Appendix Table 2).

However, some these associations reported in the fixed-effects models may have arisen because of potential time-varying associations between some of the stressors and CMD over the period. In order to explore whether the effect of the stressors on CMD changed over the months, we analysed a random-effects multilevel model, taking into account the clustering of the monthly panel observations on CMD and related stressors at the individual and primary sampling unit (PSU) levels. The coefficients from these models are detailed in Appendix Table 2. There was some evidence of the time-varying nature of the association between some of the stressors and CMD, which is illustrated in Figs 2 and 3. In both sets of figures, there was a noticeable trend of a decline in the predicted probabilities of having a CMD for nearly all the stressor groups from April to July. However, there were differences in the rate of decline (indicated by the statistically significant interactions between month and the specific stressor in Appendix Table 2). There was a steeper rate of decline in the prevalence of CMD for adults who did not report any symptoms compared to those who reported at least one symptom. People who were tested for COVID-19 in April were much more likely to have a CMD compared to those who tested in July. Compared to all other treatment groups, adults who did not have any planned healthcare treatments had a steeper decline in their probability of CMD from April to July. There was a decline in the probabilities of having CMD from April to July for nearly all the employment groups with the exception of adults who were unemployed, made redundant or had their hours reduced – this group had the highest probability (30%) of having a CMD in July compared to all the other employment groups. There was a decline in the probability of having a CMD for adults with no childcare or home schooling responsibilities and those who spent more than 16 h a week on those tasks. But for adults who spent 1–15 h a week on childcare or home schooling, there was no decline in their probability of CMD from April to June. There was no evidence that the effect of the financial stressors, loneliness or working from home on CMD differed across the months (Appendix Table 2).

## Discussion

The prevalence of CMD was highest in April 2020 with more than one in three adults living in the UK reporting problematic levels of mental health. This suggests that there was an initial shock of lockdown on CMD in April. However, as the lockdown restrictions were lifted from May onwards, the prevalence of CMD reduced steadily and by July around one in four adults had a CMD. This decreasing trend in the prevalence of CMD was mirrored by a marked decrease in the percentage of new cases of CMD in April compared to later months while the percentage of adults who recovered from a CMD was similar in April and July. We also found strong evidence of a reduction in COVID-19 and lockdown-related stressors from April to July. Most COVID-19, employment, financial and psychological ‘shocks’ were at their highest levels in April and reduced steadily in later months.

Results from this longitudinal analysis of the incidence of CMD in the UK adult population from April to July 2020 is strongly corroborated by the repeated cross-sectional surveys from the ONS Opinions and Lifestyles survey for Great Britain, which found that levels of anxiety decreased considerably and steadily since the 20th of March 2020 from nearly half of the population to 28% on the 21st of June (Davies, 2020). Furthermore, The UCL COVID-19 social study of 90 000 UK adults found that levels of anxiety and depression fell in early June as lockdown measures began to lift (Fancourt et al., 2020a, 2020b).

The novelty of this study lies in the analysis of the effects of different stressors on CMD and whether those associations differed on a monthly basis from April to July 2020. Previous studies have not been able to analyse similar monthly data where there have been large changes in potential stressors and mental health. As the pandemic and lockdown progressed, differences in the associations between some of the stressors and mental health emerged. Despite the lifting of many lockdown conditions by July and a decrease in the levels of many of the psychological and social stressors, these stressors continued to influence CMD among people who were lonely and those who were made unemployed or redundant, had financial problems or had childcare or home schooling duties.

Adults who reported COVID-19 symptoms were about 1.6–2.0 times more likely to develop CMD compared to those who did not report any symptoms. This association decreased from April to July for both those with and without any symptoms, although the decrease was markedly slower for those reporting symptoms. The association between COVID-19 symptoms and CMD is unlikely to be a consequence of having the disease as the association between testing positive for the virus and CMD decreased considerably between April and July. It is possible that worries about being infected by the virus peaked in April. There is some evidence that COVID-19 infection predicts future psychiatric disorders (Taquet, Luciano, Geddes, & Harrison, 2020), although the same study also reported associations going the other way, suggesting that the relationship between COVID-19 and mental health is complex and bidirectional.

Some of the hypothesised stressors were not associated with CMD. There did not appear to be an effect of having planned healthcare treatments cancelled or postponed on mental health in comparison to those who had their treatments as scheduled, although those who had no treatments scheduled had the lowest odds of CMD. We also found that the immediate problems of paying bills were not associated with CMD, although broader financial concerns, both currently and expected in the future, had an effect on CMD and this association was similar across the months. This finding contrasted with the results from the UCL COVID-19 Social Study (Wright et al., 2020), which found higher associations for the relationship between inability to pay bills and mental health than loss of income and mental health. It is possible that immediate concerns about paying bills were moderated to some extent by the furlough scheme which prevented some employees from becoming unemployed, although the anticipation of financial adversities in the future, perhaps in terms of future risks of unemployment clearly influenced CMD. The odds of being made unemployed or redundant on CMD in the period up to July was over twice as large as the odds for the self-employed whose businesses were not affected by the pandemic. Moreover, there was a marked increase in the probability of CMD for the unemployed in July compared to in June. In contrast, the probability of CMD for employees who were in furlough steadily decreased from April onwards. Within the self-employed group, those whose businesses were affected by the pandemic had a much higher probability of CMD in April compared to those whose businesses were not affected; but by July, there were no differences between these two self-employed groups. This may have been because of the relaxation of lockdown restrictions on most businesses in July, allowing many small businesses to reopen. As unemployment and redundancy increase in the labour market, it will be important to keep monitoring the mental health consequences of unemployment. Employees who were furloughed had about the same levels of incident CMD as employees whose job hours were not affected. This suggests that the government measures to protect jobs also had positive mental health benefits for those employees who were able to keep their jobs albeit in a ‘furloughed’ state.

Adults who were always working from home had the highest odds of CMD, suggesting there may be stressors associated with home working. An example of this was the finding that spending more time on childcare or home schooling was also associated with a small increased risk of CMD, at least until June. Loneliness was the largest predictor of CMD and this association remained similar between April and July. While the effect of loneliness on developing CMD is unsurprising, the size of the effect (an odds ratio of 16 times comparing those often lonely to those hardly lonely) is remarkable. Even though the prevalence of those who were often lonely decreased a little from April to June, the fact that nearly 7% of the adult population reported often feeling lonely in July is of concern.

This is the first population representative study in the UK that analyses longitudinal changes in the mental health of UK adults in relation to changes in stressors arising from the pandemic and lockdown conditions from April to July 2020. Adults from across the entire adult age range were analysed with detailed measures of psychological, social and economic stressors. Although the measure of CMD was self-reported, the GHQ-12 has been validated in a number of studies (Goldberg & Williams, 1988; Goldberg et al., 1997). Loneliness and CMD were self-reported, and some of this association may be due to common method variance. However, the fixed-effects regression models analyse within-person change, which reduces the bias associated with self-reported measures. The w10–11 interviews were conducted face-to-face, on the web and by telephone; the COVID-19 surveys were solely carried out online, so there may be mode effects. Davillas and Jones tested for this in the analyses of April data and found no significant mode effects compared to the w9 interviews (Davillas & Jones, 2020). The UKHLS data are not linked to COVID-19 testing and results, so we relied on self-reports from study participants, which could underestimate the effects of COVID-19 on mental health.

The measure of mental health was self-reported and pertained to CMD and not major psychiatric conditions. There may be differing patterns for those with more severe mental health problems. A longitudinal study on mental health and wellbeing in the UK from the end of March to 11 May 2020 reported an increase in suicidal ideation over the period, whereas symptoms of anxiety, levels of defeat and entrapment decreased over the same period and positive wellbeing increased (O'Connor et al., 2020).

Loneliness was the major determinant of CMD during lockdown among adults in the UK. Subsequent to April 2020, furloughing has been effective in mitigating the increase in CMD for some groups of employees. Although the incidence of CMD reduced to pre-pandemic levels by July 2020, the risk to CMD of becoming unemployed or redundant was evident by July. Despite some reduction in levels of stressors by the middle of 2020, an increase in unemployment as the recession unfolds and related financial stressors are also likely to lead to increased levels of CMD.

## Supplementary material

For supplementary material accompanying this paper visit https://doi.org/10.1017/S0033291720005048. click here to view supplementary material

## Partial patient and public involvement (PPI) statement

Some of the study participants provided feedback on the first COVID19 survey which we took into account in the design of the second and subsequent surveys. We did not directly include PPI in this study, but there is an active dissemination and communication strategy with the participants. They receive a newsletter of findings four times a year, and we keep a participant website up to date.
