# Supplementary material for: The mental health impact of COVID-19 and lockdown-related stressors among adults in the UK
Source: Psychol Med. 2020 Dec 7:1–10. doi: 10.1017/S0033291720005048 (PMC7783135; doi:10.1017/S0033291720005048)
Supplement: Supplementary file 1 [file S0033291720005048sup001.docx]

Appendix Table 1: Distribution of key socio-demographic and health factors in the April survey

|  | **April** | *April n* |
| --- | --- | --- |
| **Age-group** |  |  |
| 15-19 | 2.6% | *361* |
| 20-24 | 7.9% | *1,101* |
| 25-29 | 8.0% | *1,115* |
| 30-34 | 6.0% | *834* |
| 35-39 | 7.9% | *1,107* |
| 40-44 | 7.0% | *981* |
| 45-49 | 8.7% | *1,219* |
| 50-54 | 9.1% | *1,275* |
| 55-59 | 10.0% | *1,391* |
| 60-64 | 8.7% | *1,209* |
| 65-69 | 7.3% | *1,023* |
| 70-74 | 7.5% | *1,050* |
| 75+ | 9.3% | *1,306* |
| **Sex** |  |  |
| Male | 48.0% | *6,706* |
| Female | 52.0% | *7,252* |
| **Living with a partner** |  |  |
| Yes | 63.3% | *8,843* |
| No | 36.7% | *5,131* |
| **Living with children under 5** |  |  |
| no child under 5 | 91.0% | *12,715* |
| 1 or more child under 5 | 9.0% | *1,259* |
| **Ethnicity** |  |  |
| White British/Irish | 88.5% | *12,205* |
| Other White ethnicity | 2.7% | *377* |
| Indian | 2.0% | *271* |
| Pakistani/Bangladeshi | 1.9% | *262* |
| Black Caribbean/African | 1.8% | *254* |
| Mixed | 1.5% | *211* |
| Chinese/other Asian/Arab/Other | 1.6% | *214* |
| **Qualifications** |  |  |
| Degree | 29.4% | *3,912* |
| Other higher degree | 11.8% | *1,577* |
| A-level etc | 22.8% | *3,033* |
| GCSE etc | 20.5% | *2,737* |
| Other qualifications | 9.2% | *1,227* |
| No qualifications | 6.3% | *842* |
| **Shielding status** |  |  |
| No letter/text received | 92.3% | *12,882* |
| Shielding letter/text received | 7.7% | *1,069* |
| **Chronic health conditions** |  |  |
| None | 51.2% | *7,099* |
| 1 | 28.2% | *3,913* |
| 2 or more | 20.5% | *2,841* |

Appendix Table 2: Multilevel logistic regression models predicting common mental disorder

|  | **Apr-Jul** | **Apr-June** | **Apr-Jul (no June)** |
| --- | --- | --- | --- |
| **Main effects** | ***p<0.01*** | ***p<0.01*** | ***p<0.01*** |
| **Intercept** | -1.99 (0.29) | -1.84 (0.29) | -1.96 (0.29) |
| **Survey month (ref:April)** | ***p<0.05*** | *p>0.05* | *p>0.05* |
| May | 0.21 (0.22) | 0.23 (0.22) | 0.04 (0.26) |
| June | -0.03 (0.23) | -0.03 (0.22) |  |
| July | -0.47 (0.25) |  | -0.43 (0.30) |
| **Time since w9 interview** | *p>0.05* | *p>0.05* | *p>0.05* |
| Months | -0.003 (0.004) | -0.003 (0.004) | 0.001 (0.003) |
| **Age-group (ref: 15-19)** | ***p<0.01*** | ***p<0.01*** | ***p<0.01*** |
| 20-24 | 0.1 (0.23) | -0.01 (0.24) | -0.08 (0.22) |
| 25-29 | -0.06 (0.23) | -0.27 (0.24) | -0.38 (0.23) |
| 30-34 | -0.06 (0.23) | -0.26 (0.24) | -0.44 (0.22) |
| 35-39 | -0.08 (0.22) | -0.28 (0.23) | -0.50 (0.22) |
| 40-44 | -0.05 (0.22) | -0.25 (0.23) | -0.53 (0.21) |
| 45-49 | -0.07 (0.21) | -0.30 (0.22) | -0.59 (0.21) |
| 50-54 | -0.15 (0.22) | -0.32 (0.23) | -0.58 (0.21) |
| 55-59 | -0.26 (0.21) | -0.41 (0.22) | -0.54 (0.21) |
| 60-64 | -0.46 (0.22) | -0.55 (0.23) | -0.73 (0.21) |
| 65-69 | -0.70 (0.22) | -0.79 (0.23) | -0.81 (0.22) |
| 70-74 | -0.86 (0.23) | -0.87 (0.23) | -0.88 (0.22) |
| 75+ | -0.86 (0.23) | -0.91 (0.23) | -0.74 (0.22) |
| **Sex (ref: male)** | ***p<0.01*** | ***p<0.01*** | ***p<0.01*** |
| Female | 0.59 (0.05) | 0.58 (0.05) | 0.58 (0.05) |
| **Ethnicity (ref: White British/Irish)** | ***p<0.05*** | ***p<0.05*** | ***p<0.01*** |
| Other White ethnicity | -0.01 (0.17) | 0.05 (0.16) | -0.04 (0.15) |
| Indian | -0.11 (0.18) | -0.01 (0.18) | -0.40 (0.16) |
| Pakistani/Bangladeshi | -0.51 (0.18) | -0.31 (0.18) | -0.80 (0.17) |
| Black Caribbean/African | -0.59 (0.24) | -0.59 (0.23) | -0.89 (0.21) |
| Mixed | 0.21 (0.21) | 0.25 (0.19) | 0.05 (0.18) |
| Other | -0.39 (0.23) | -0.40 (0.21) | -0.52 (0.23) |
| **Living with a partner (yes)** | ***p<0.01*** | ***p<0.01*** | ***p<0.01*** |
| No | -0.18 (0.06) | -0.21 (0.06) | -0.33 (0.06) |
| **Living with children under 5 (ref: no)** | *p>0.05* | *p>0.05* | *p>0.05* |
| Yes | 0.19 (0.10) | 0.03 (0.11) | 0.12 (0.10) |
| **Qualifications (ref: degree)** | ***p<0.01*** | ***p<0.01*** | ***p<0.01*** |
| Other higher degree | -0.21 (0.08) | -0.19 (0.08) | -0.24 (0.07) |
| A-level etc | -0.29 (0.07) | -0.27 (0.07) | -0.38 (0.07) |
| GCSE etc | -0.29 (0.08) | -0.27 (0.07) | -0.39 (0.07) |
| Other qualification | -0.56 (0.12) | -0.58 (0.12) | -0.69 (0.12) |
| No qualification | -0.86 (0.15) | -0.82 (0.14) | -0.94 (0.14) |
| **Chronic health conditions** | ***p<0.01*** | ***p<0.01*** | ***p<0.01*** |
| 1 | 0.17 (0.06) | 0.13 (0.06) | 0.16 (0.05) |
| 2 or more | 0.43 (0.07) | 0.36 (0.07) | 0.29 (0.07) |
| **Shielding status (ref: not shielding)** | *p>0.05* | *p>0.05* | *p>0.05* |
| Shielding | 0.14 (0.11) | 0.17 (0.11) | 0.06 (0.10) |
|  | **Apr-Jul** | **Apr-June** | **Apr-Jul (no June)** |
| **Reported COVID-19 symptoms (ref: none)** | p<0.05 | p<0.05 | p<0.05 |
| At least one | 0.20 (0.09) | 0.20 (0.09) | 0.18 (0.09) |
| Reported COVID-19 test (ref: no test) | p<0.01 | p<0.01 | p<0.01 |
| **Tested for COVID-19** | 0.98 (0.30) | 1.00 (0.31) | 0.83 (0.33) |
| Tested positive | 1.91 (0.62) | 2.10 (0.68) | 1.72 (0.64) |
| **Health treatments (ref: no treatments planned)** | p<0.01 | p>0.05 | p>0.05 |
| treatments cancelled/postponed | 0.12 (0.12) | 0.19 (0.11) | 0.17 (0.09) |
| I cancelled treatments | 0.27 (0.22) | 0.34 (0.21) | 0.30 (0.22) |
| alternative treatment/scheduled | 0.13 (0.16) | 0.12 (0.16) | 0.13 (0.14) |
| **Employment Status (ref: slf emplyd unaffected)** | p<0.01 | p<0.01 | p<0.01 |
| self-employed- -vely impacted by COVID | 0.80 (0.25) | 0.91 (0.23) | 0.54 (0.23) |
| employee- hours not affected | 0.41 (0.20) | 0.45 (0.19) | 0.64 (0.19) |
| employee- redundant/unemp/reduced hours | 0.76 (0.24) | 0.82 (0.24) | 0.74 (0.23) |
| employee- furloughed | 0.40 (0.23) | 0.43 (0.23) | 0.46 (0.22) |
| self-employed and employee-self-isolating/caring | 0.83 (0.23) | 0.79 (0.23) | 0.82 (0.23) |
| not in work Jan/Feb | 0.70 (0.25) | 0.68 (0.24) | 0.93 (0.22) |
| **How often working from home (ref: always)** | p<0.01 | p<0.05 | p<0.01 |
| Often | -0.08 (0.14) | -0.14 (0.14) | -0.01 (0.13) |
| Sometimes | -0.24 (0.15) | -0.20 (0.14) | -0.29 (0.14) |
| Never | -0.34 (0.12) | -0.37 (0.11) | -0.41 (0.11) |
| No paid work hours | -0.23 (0.13) | -0.23 (0.13) | -0.45 (0.12) |
| **How often feel lonely (ref: Hardly/Never)** | p<0.01 | p<0.01 | p<0.01 |
| Some of the time | 2.06 (0.12) | 2.03 (0.11) | 1.90 (0.08) |
| Often | 3.84 (0.20) | 3.67 (0.17) | 3.45 (0.19) |
| **Hrs/week on chldcare (ref: no chld<18/0 hrs)** |  | p<0.01 |  |
| 1-15 hours/week |  | 0.04 (0.11) |  |
| 16 hours or more/week |  | 0.42 (0.12) |  |
| **Problems paying bills from last survey (ref: no problems)** |  |  | p>0.05 |
| Behind with some bills |  |  | 0.38 (0.21) |
| Behind with all bills |  |  | 0.46 (0.88) |
| **Subjective Financial situation (ref: comfortable)** |  |  | p<0.01 |
| Doing alright |  |  | 0.42 (0.08) |
| Just about getting by |  |  | 0.85 (0.10) |
| Finding it quite/very difficult |  |  | 1.51 (0.18) |
| **Future expectation finances(ref: better off)** |  |  | p<0.01 |
| Worse off than now |  |  | 0.75 (0.17) |
| or about the same? |  |  | -0.08 (0.11) |

| **May Interaction effects** | **Apr-Jul** | **Apr-June** | **Apr-Jul (no June)** |
| --- | --- | --- | --- |
| **Reported COVID-19 symptoms*** | ***p<0.01*** | ***p<0.01*** | ***p<0.05*** |
| At least one | 0.48 (0.21) | 0.38 (0.19) | 0.39 (0.20) |
| **Reported COVID-19 test*** | ***p<0.05*** | ***p<0.01*** | ***p<0.01*** |
| Tested for COVID-19 | -0.99 (0.33) | -1.03 (0.34) | -0.91 (0.35) |
| Tested positive | -2.92 (1.09) | -3.09 (1.13) | -2.39 (1.03) |
| **Health treatments*** | ***p<0.05*** | ***p<0.05*** | *p>0.05* |
| treatments cancelled/postponed | 0.34 (0.20) | 0.36 (0.19) | 0.22 (0.14) |
| I cancelled treatments | 0.47 (0.36) | 0.28 (0.34) | 0.30 (0.33) |
| alternative treatment/scheduled | 0.19 (0.25) | 0.24 (0.23) | 0.14 (0.20) |
| **Employment Status*** | *p>0.05* | *p>0.05* | *p>0.05* |
| self-employed- -vely impacted by COVID | -0.84 (0.39) | -0.93 (0.36) | -0.53 (0.42) |
| employee- hours not affected | -0.54 (0.22) | -0.59 (0.22) | -0.53 (0.21) |
| employee- redundant/unemp/reduced hours | -0.51 (0.37) | -0.59 (0.36) | -0.32 (0.37) |
| employee- furloughed | -0.6 (0.33) | -0.58 (0.34) | -0.67 (0.3) |
| self-employed and employee-self-isolating/caring | -0.38 (0.35) | -0.34 (0.35) | -0.11 (0.35) |
| not in work Jan/Feb | -0.94 (0.33) | -0.96 (0.32) | -0.73 (0.25) |
| **How often working from home*** | *p>0.05* | *p>0.05* | *p>0.05* |
| Often | 0.17 (0.2) | 0.16 (0.20) | 0.05 (0.19) |
| Sometimes | -0.31 (0.21) | -0.27 (0.20) | -0.33 (0.20) |
| Never | -0.09 (0.15) | -0.06 (0.15) | -0.14 (0.15) |
| No paid work hours | 0.24 (0.16) | 0.23 (0.16) | 0.14 (0.15) |
| **How often feel lonely*** | *p>0.05* | *p>0.05* | *p>0.05* |
| Some of the time | -0.10 (0.22) | -0.05 (0.21) | 0.03 (0.12) |
| Often | 0.18 (0.29) | 0.28 (0.25) | 0.23 (0.26) |
| **Hours/week on childcare/home schooling*** |  | *p>0.05* |  |
| 1-15 hours/week |  | -0.03 (0.14) |  |
| 16 hours or more/week |  | 0.08 (0.14) |  |
| **Problems paying bills*** |  |  | *p>0.05* |
| Behind with some bills |  |  | 0.28 (0.24) |
| Behind with all bills |  |  | -0.35 (1.05) |
| **Subjective Financial situation*** |  |  | *p>0.05* |
| Doing alright |  |  | 0.08 (0.10) |
| Just about getting by |  |  | 0.05 (0.15) |
| Finding it difficult |  |  | -0.12 (0.26) |
| **Future expectation finances*** |  |  | *p>0.05* |
| Worse off than now |  |  | 0.38 (0.23) |
| or about the same? |  |  | 0.12 (0.16) |

| **June Interaction effects** | **Apr-Jul** | **Apr-June** | **Apr-Jul (no June)** |
| --- | --- | --- | --- |
| **Reported COVID-19 symptoms*** | *p>0.05* | *p>0.05* |  |
| At least one | 0.14 (0.26) | 0.22 (0.25) |  |
| **Reported COVID-19 test*** | *p>0.05* | *p>0.05* |  |
| Tested for COVID-19 | -0.82 (0.35) | -0.83 (0.36) |  |
| Tested positive | -0.92 (1.06) | -1.25 (1.04) |  |
| **Health treatments*** | ***p<0.01*** | ***p<0.01*** |  |
| treatments cancelled/postponed | 0.22 (0.17) | 0.23 (0.15) |  |
| I cancelled treatments | 0.10 (0.38) | 0.16 (0.37) |  |
| alternative treatment/scheduled | 0.77 (0.29) | 0.64 (0.21) |  |
| **Employment Status*** | ***p<0.01*** | ***p<0.01*** |  |
| self-employed- -vely impacted by COVID | -0.08 (0.69) | -0.16 (0.66) |  |
| employee- hours not affected | -0.67 (0.23) | -0.62 (0.22) |  |
| employee- redundant/unemp/reduced hours | -1.62 (0.50) | -1.65 (0.47) |  |
| employee- furloughed | -1.08 (0.37) | -1.06 (0.37) |  |
| self-employed and employee-self-isolating/caring | -0.26 (0.44) | -0.05 (0.43) |  |
| not in work Jan/Feb | -0.95 (0.28) | -1.07 (0.27) |  |
| **How often working from home*** | *p>0.05* | *p>0.05* |  |
| Often | 0.14 (0.22) | 0.12 (0.21) |  |
| Sometimes | 0.29 (0.21) | 0.19 (0.20) |  |
| Never | 0.03 (0.18) | 0.02 (0.17) |  |
| No paid work hours | 0.36 (0.19) | 0.42 (0.17) |  |
| **How often feel lonely*** | *p>0.05* | *p>0.05* |  |
| Some of the time | 0.16 (0.16) | 0.09 (0.13) |  |
| Often | 0.43 (0.36) | 0.51 (0.32) |  |
| **Hours/week on childcare/home schooling*** |  | ***p<0.01*** |  |
| 1-15 hours/week |  | 0.21 (0.14) |  |
| 16 hours or more/week |  | -0.23 (0.15) |  |
| **Problems paying bills*** |  |  |  |
| Behind with some bills |  |  |  |
| Behind with all bills |  |  |  |
| **Subjective Financial situation*** |  |  |  |
| Doing alright |  |  |  |
| Just about getting by |  |  |  |
| Finding it different |  |  |  |
| **Future expectation finances*** |  |  |  |
| Worse off than now |  |  |  |
| or about the same? |  |  |  |

| **July Interaction effects** | **Apr-Jul** | **Apr-June** | **Apr-Jul (no June)** |
| --- | --- | --- | --- |
| **Reported COVID-19 symptoms*** |  |  | ***p<0.01*** |
| At least one | 0.31 (0.27) |  | 0.62 (0.27) |
| **Reported COVID-19 test*** | *p>0.05* |  | ***p<0.05*** |
| Tested for COVID-19 | -0.88 (0.35) |  | -0.95 (0.41) |
| Tested positive | -1.89 (1.12) |  | -1.69 (1.02) |
| **Health treatments*** | ***p<0.01*** |  | ***p<0.05*** |
| treatments cancelled/postponed | 0.52 (0.2) |  | 0.42 (0.17) |
| I cancelled treatments | -0.74 (1.15) |  | -0.86 (1.02) |
| alternative treatment/scheduled | 0.46 (0.21) |  | 0.29 (0.19) |
| **Employment Status*** | ***p<0.01*** |  | ***p<0.01*** |
| self-employed- -vely impacted by COVID | -0.78 (0.48) |  | -0.66 (0.46) |
| employee- hours not affected | -0.67 (0.25) |  | -0.72 (0.23) |
| employee- redundant/unemp/reduced hours | 0.10 (0.37) |  | -0.04 (0.36) |
| employee- furloughed | -1.25 (0.58) |  | -1.10 (0.57) |
| self-employed and employee-self-isolating/caring | -0.70 (0.57) |  | -0.42 (0.52) |
| not in work Jan/Feb | -1.11 (0.31) |  | -1.03 (0.29) |
| **How often working from home*** | *p>0.05* |  | *p>0.05* |
| Often | -0.09 (0.23) |  | -0.10 (0.22) |
| Sometimes | 0.23 (0.22) |  | 0.24 (0.20) |
| Never | -0.05 (0.16) |  | -0.14 (0.15) |
| No paid work hours | 0.53 (0.20) |  | 0.43 (0.18) |
| **How often feel lonely*** | *p>0.05* |  | *p>0.05* |
| Some of the time | -0.12 (0.15) |  | -0.08 (0.12) |
| Often | 0.32 (0.33) |  | 0.55 (0.34) |
| **Problems paying bills*** |  |  | *p>0.05* |
| Behind with some bills |  |  | 0.39 (0.27) |
| Behind with all bills |  |  | -0.08 (1.04) |
| **Subjective Financial situation*** |  |  | *p>0.05* |
| Doing alright |  |  | -0.12 (0.12) |
| Just about getting by |  |  | -0.03 (0.16) |
| Finding it different |  |  | 0.39 (0.32) |
| **Future expectation finances*** |  |  | *p>0.05* |
| Worse off than now |  |  | -0.01 (0.29) |
| or about the same? |  |  | 0.17 (0.19) |
|  |  |  |  |
| **Random Part (variances)** |  |  |  |
| PSU variance (level 3) | 0.45 | 0.44 | 0.37 |
| Individual variance (Level 2) | 1.71 | 1.23 | 0.93 |
| Month variance (level 1) | 1 | 1 | 1 |
| Units: PSU | 3529 | 3506 | 3513 |
| Units: Individual | 12553 | 12372 | 12389 |
| Units: Month | 43104 | 32715 | 32504 |
